# Supplementary material for: Intrinsic Interferon Signaling Regulates the Cell Death and Mesenchymal Phenotype of Glioblastoma Stem Cells
Source: Cancers (Basel). 2021 Oct 21;13(21):5284. doi: 10.3390/cancers13215284 (PMC8582372; doi:10.3390/cancers13215284)
Supplement: Supplementary file 1 [file cancers-13-05284-s001.zip › cancers-1435500-supplementary.pdf]

cancers

Supplementary Materials: Intrinsic Interferon Signaling Regulates the Cell Death and Mesenchymal Phenotype of Glioblastoma Stem Cells

Sabbir Khan, Rajasekaran Mahalingam, Shayak Sen, Emmanuel Martinez-Ledesma, Arshad Khan, Kaitlin Gandy, Frederick F. Lang, Erik P. Sulman, Kristin D. Alfaro-Munoz, Nazanin K. Majd, Veerakumar Balasubramaniyan and John F. de Groot

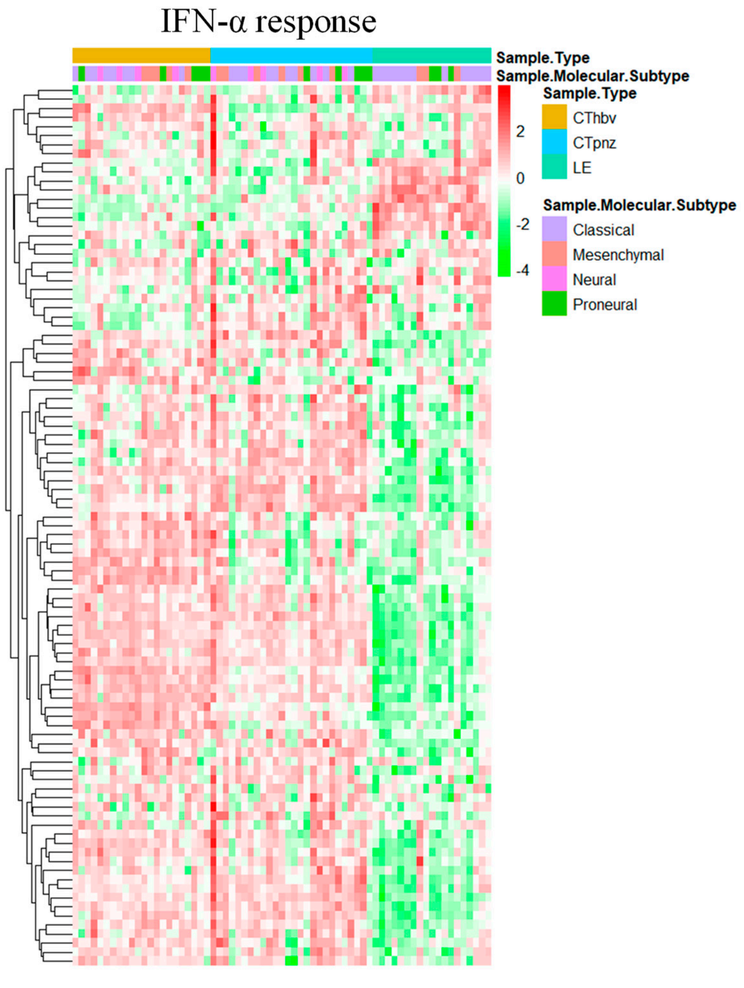

Figure S1. Analysis of type I and type II IFN signaling genes expression in different compartments of GBM patients' tumors. The normalized gene-level FPKM matrix for RNA-seq data was obtained from the Ivy Glioblastoma Atlas Project (https://glioblastoma.alleninstitute.org/). The matrix was converted into a z-score for plotting the selected genes. Heat maps were generated using the pheatmap R package [1]. The molecular subtypes of GBM patients are classified by Verhaak et al. [2]. CTpnz: perinecrotic zone, CThbv: hyperplastic blood vessels in the cellular tumor, and LE: leading edge.

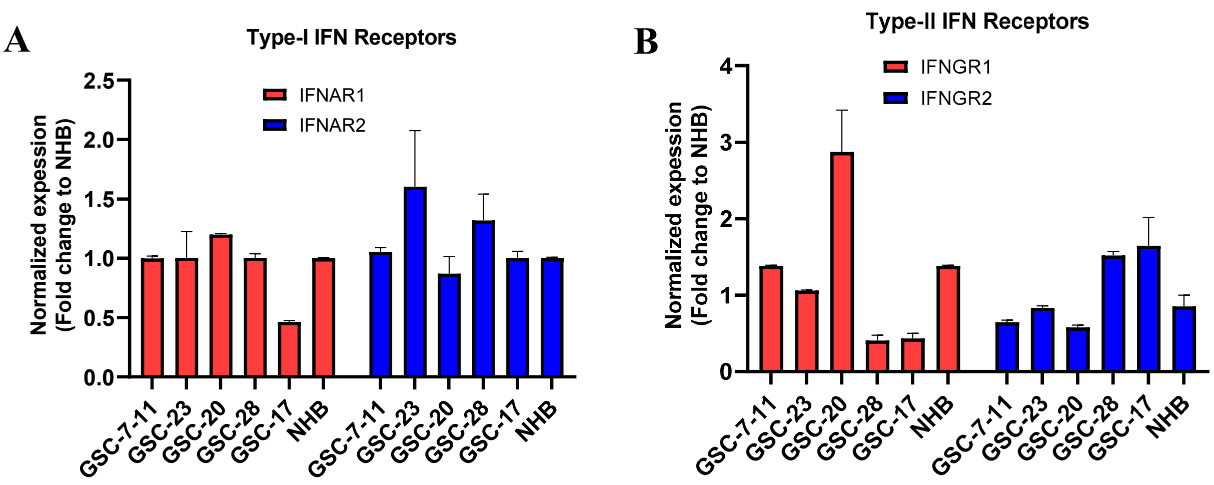

Figure S2. Basal expression of type I and type II IFN signaling receptors in a cohort of GSCs. The mRNA expression of IFNAR1, and IFNAR2; IFNGR1, and IFNGR2 in a cohort of GSCs using qPCR, and the expression was normalized with normal huma brain (NHB) RNA.

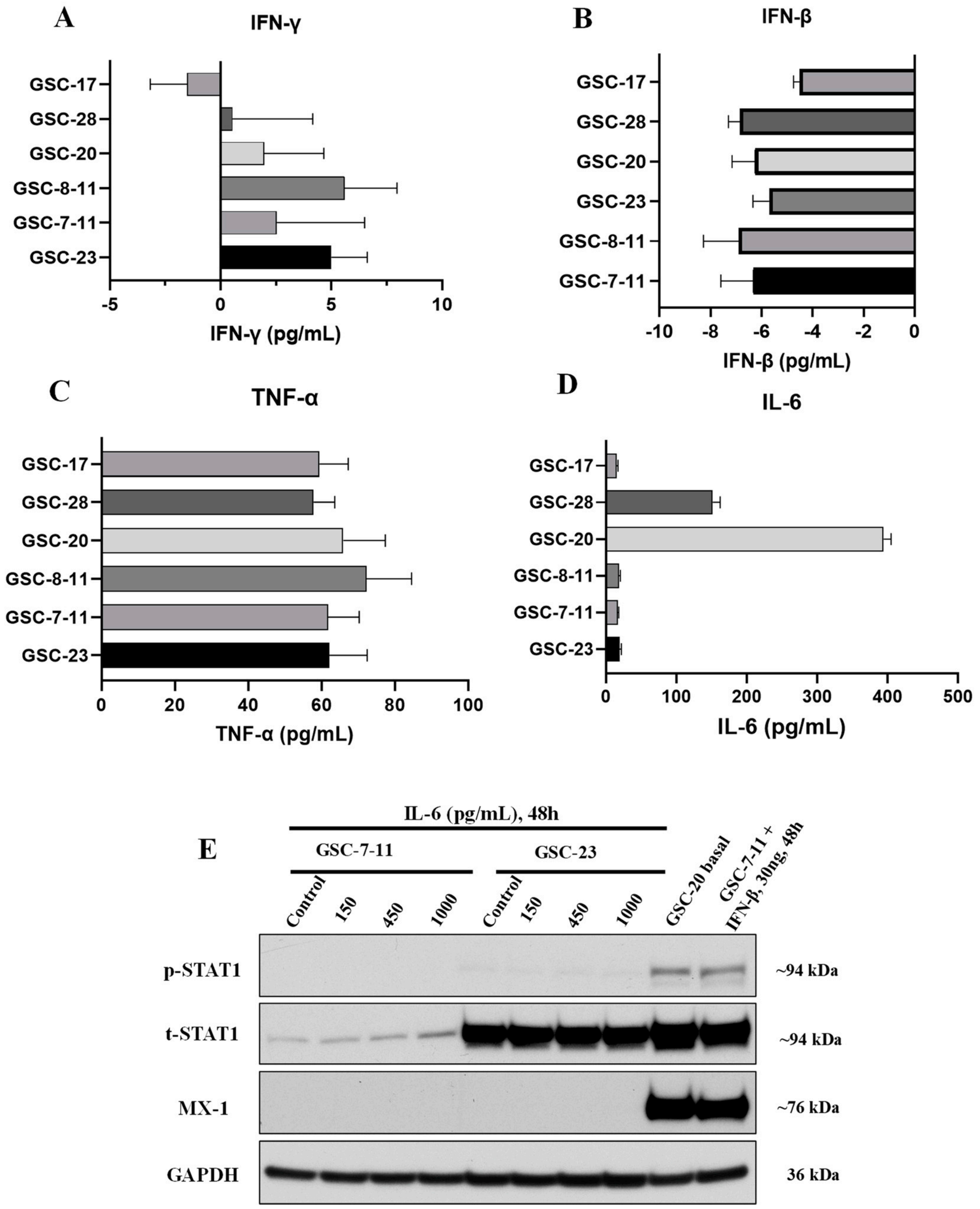

Figure S3. Secretion of IFNs and inflammatory cytokines in the cell culture media under basal conditions in GSCs and western blot analysis of IL-6 treated intrinsically low IFN GSCs. (A-D) the secretion of IFN-γ, IFN-β, and inflammatory cytokines TNF-α and IL-6 in CM from intrinsically low and and high IFN signaling GSCs evaluated using ELISA assays. (E) Representative WB of IFN/STAT1 signaling proteins in whole-cell lysates of GSC-7-11 and GSC-23 treated with human recombinant IL-6 (150-1000 pg/mL) for 48h. GSC-20 basal who;e cell lysate and GSC-7-11 cells treated with 30 ng/mL for 48h whole cell lysates were used as positive control.

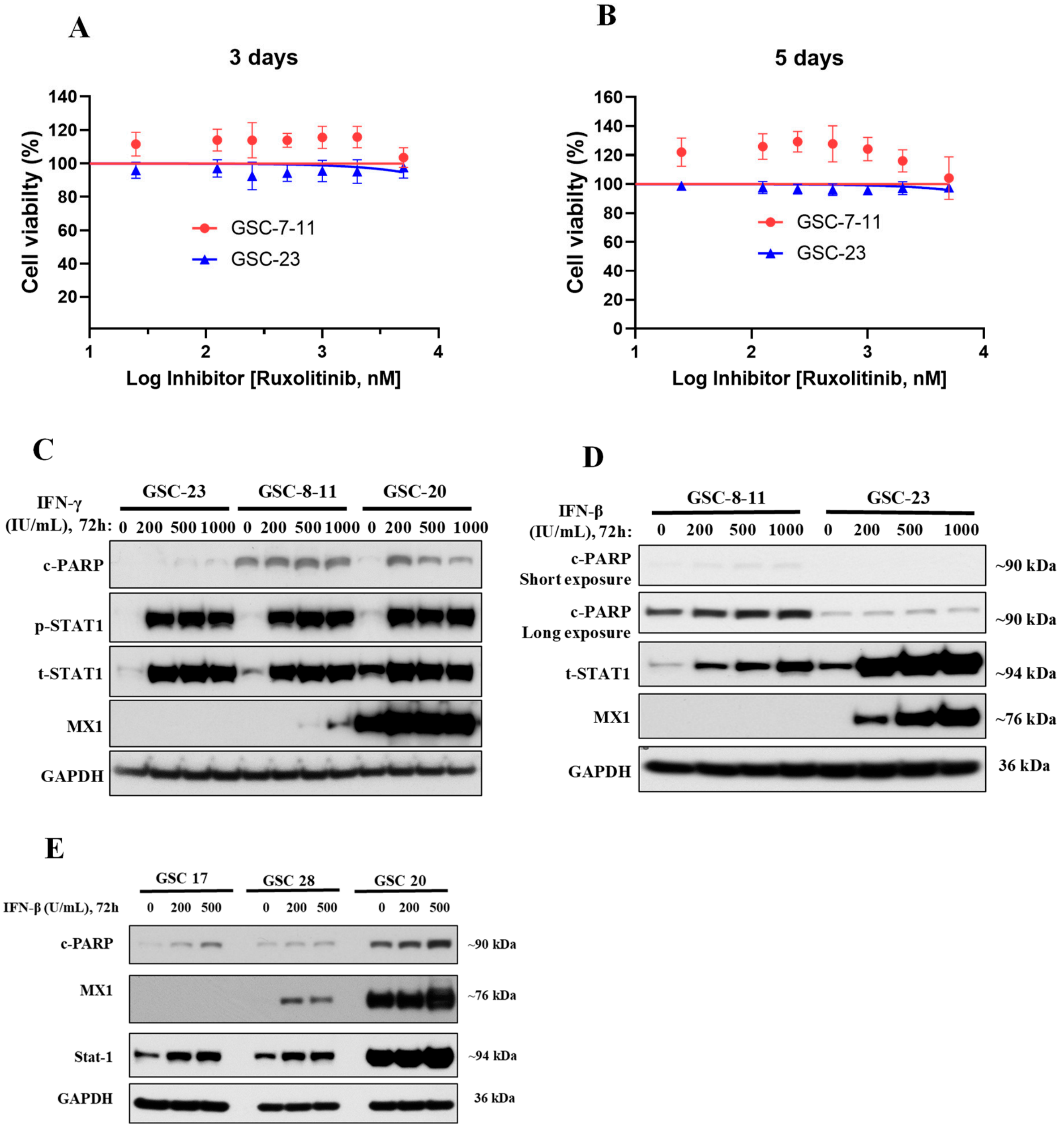

Figure S4. Cell proliferation of intrinsically low IFN GSCs treated with ruxolitinib for 3-5 days, and western blots analysis in a cohort of GSC treated with IFN-γ and IFN-β. (A and B) Cell viability and proliferation of GSC-7-11, and GSC-23 treated with ruxolitinib (25-5000 nM) for 3 days and 5 days, respectively. (C) Representative WB of c-PARP (apoptosis) and IFN/STAT1 signaling proteins in whole-cell lysates of GSC-23, GSC-8-11, and GSC-20 treated with IFN-γ (200-1000 IU/mL) for 72 h. (D) WB of c-PARP and IFN/STAT1 signaling proteins in whole-cell lysates of GSC-8-11 and GSC-23 treated with IFN-β (200-1000 IU/mL) for 72h. (E) Representative WB of c-PARP and IFN/STAT1 signaling proteins in whole-cell lysates of GSC-17, GSC-28, and GSC-20 treated with IFN-β (200-500 IU/mL) for 72h.

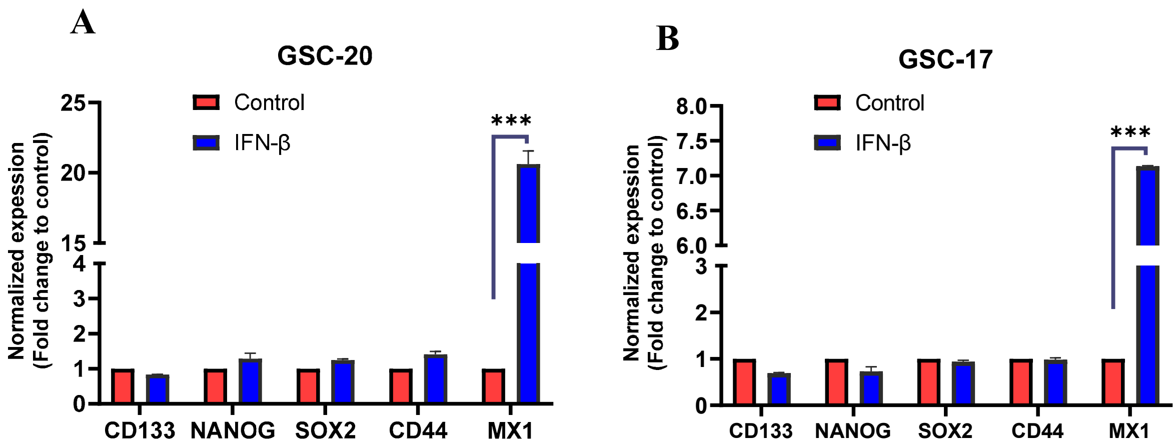

Figure S5. mRNA expression of stemness markers in GSC-20 and GSC-17 treated with IFN-β. (A and B) The mRNA expression of the stemness and IFN signature genes in GSC-20 and GSC-17 treated with IFN-β (1000 IU/mL) for 72h, respectively. *** $p < 0.001$ as compared to untreated cells.

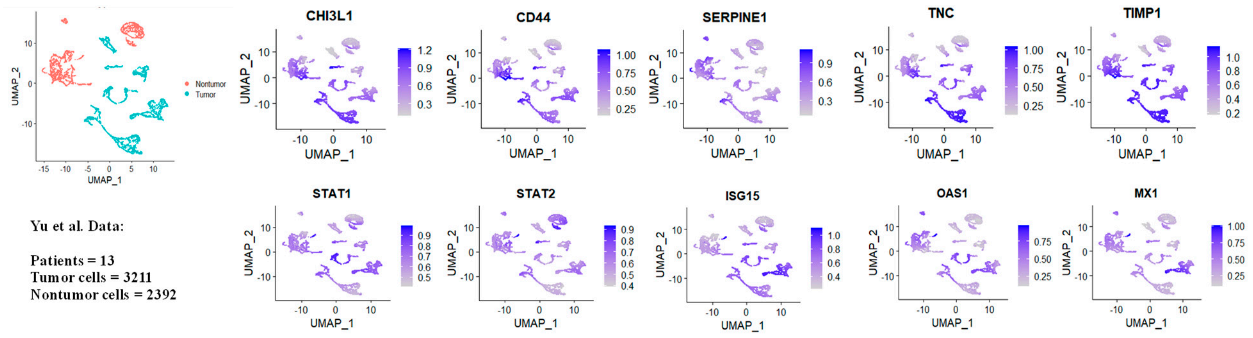

Figure S6. The scRNA-seq analysis for the selected genes for the IFN signaling, and mesenchymal signatures in tumor and nontumor cells, data obtained from the Yu et al (GSE117891) [3].

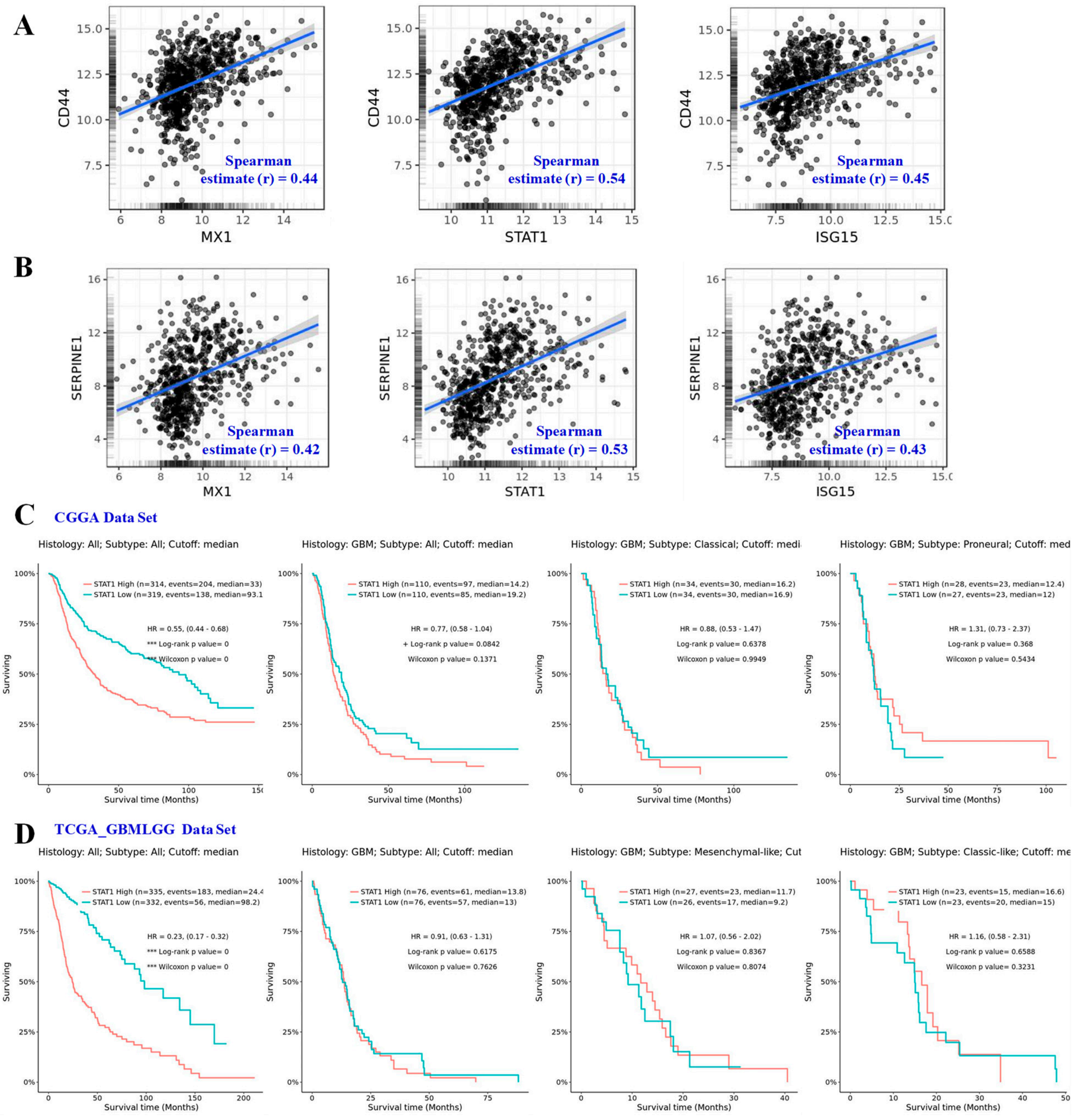

Figure S7. (A and B) Correlation analyses of *CD44* and *SERPINE1* with *MX1*, *STAT1*, and *ISG15* mRNA levels in glioma patients from the TCGA data set analyzed using the GlioVis platform. (C and D) Survival analysis of the low and high expression of *STAT1* in the mesenchymal glioma/GBM tumors specimens in the TCGA and CGGA datasets, respectively. Analyzed using the GlioVis platform.

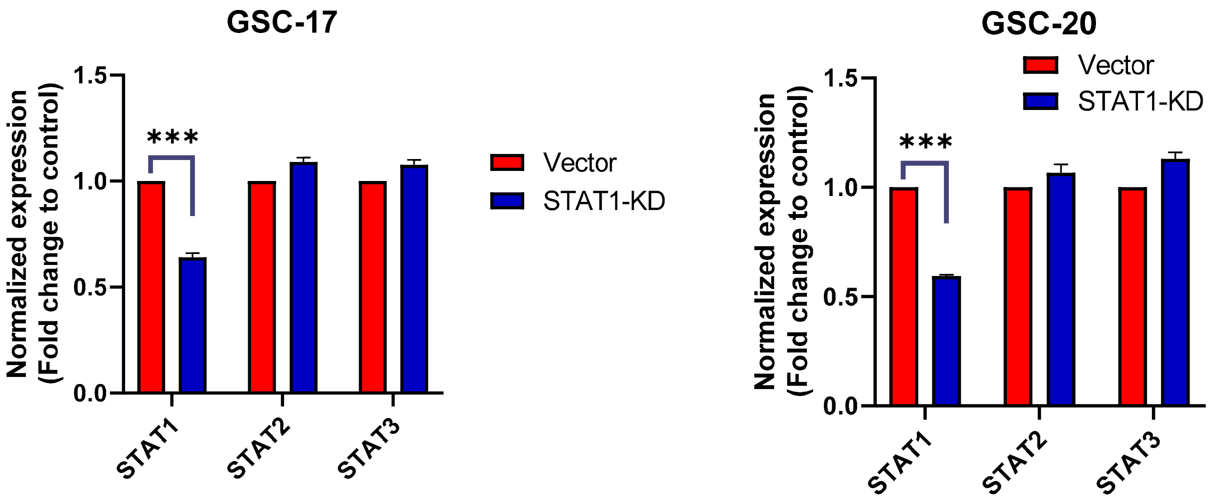

Figure S8. The basal mRNA expression of STAT1, STAT2 and STAT3 in vector control and STAT1 KD GSCs. (A and B) CRISPR/Cas9-mediated STAT1 KD specifically reduced the basal expression of STAT1 as compared to vector control in GSC17 and GSC20, respectively. STAT1 KD did not change the basal expression of STAT2 and STAT3.

References:

1. Kolde, R. pheatmap: Pretty Heatmaps. R package version 0.7.7. Available online: http://CRAN.R-project.org/package=pheatmap (accessed on 21 April 2021).
2. Verhaak, R.G.; Hoadley, K.A.; Purdom, E.; Wang, V.; Qi, Y.; Wilkerson, M.D.; Miller, C.R.; Ding, L.; Golub, T.; Mesirov, J.P.; et al. Integrated genomic analysis identifies clinically relevant subtypes of glioblastoma characterized by abnormalities in PDGFRA, IDH1, EGFR, and NF1. *Cancer Cell* **2010**, *17*, 98–110, doi:10.1016/j.ccr.2009.12.020.
3. Yu, K.; Hu, Y.Q.; Wu, F.; Guo, Q.F.; Qian, Z.H.; Hu, W.E.; Chen, J.; Wang, K.Y.; Fan, X.Y.; Wu, X.L.; et al. Surveying brain tumor heterogeneity by single-cell RNA-sequencing of multi-sector biopsies. *National Science Review* **2020**, *7*, 1306–318, doi:10.1093/nsr/nwaa099.
